# The novel BET inhibitor UM-002 reduces glioblastoma cell proliferation and invasion

**DOI:** 10.1038/s41598-021-02584-6

**Published:** 2021-12-03

**Authors:** Anna M. Jermakowicz, Matthew J. Rybin, Robert K. Suter, Jann N. Sarkaria, Zane Zeier, Yangbo Feng, Nagi G. Ayad

**Affiliations:** 1 Department of Neurological Surgery, Miami Project To Cure Paralysis, Sylvester Comprehensive Cancer Center, University of Miami Miller School of Medicine, Miami, FL 33136 USA; 2 Department of Psychiatry and Behavioral Sciences, Sylvester Comprehensive Cancer Center, University of Miami Miller School of Medicine, Miami, FL 33136 USA; 3 Department of Radiation Oncology, Mayo Clinic, Rochester, MN 55905 USA; 4 Department of Molecular and Cellular Pharmacology, Sylvester Comprehensive Cancer Center, University of Miami Miller School of Medicine, Miami, FL 33136 USA; 5 Department of Oncology, Lombardi Comprehensive Cancer Center, Georgetown University, Washington, DC, 20057 USA

**Keywords:** Cell biology, Cell invasion, Computational biology and bioinformatics, Drug discovery, Medicinal chemistry, Blood-brain barrier, Neurological models, Biological models, Cancer models

## Abstract

Bromodomain and extraterminal domain (BET) proteins have emerged as therapeutic targets in multiple cancers, including the most common primary adult brain tumor glioblastoma (GBM). Although several BET inhibitors have entered clinical trials, few are brain penetrant. We have generated UM-002, a novel brain penetrant BET inhibitor that reduces GBM cell proliferation in vitro and in a human cerebral brain organoid model. Since UM-002 is more potent than other BET inhibitors, it could potentially be developed for GBM treatment. Furthermore, UM-002 treatment reduces the expression of cell-cycle related genes in vivo and reduces the expression of invasion related genes within the non-proliferative cells present in tumors as measured by single cell RNA-sequencing. These studies suggest that BET inhibition alters the transcriptional landscape of GBM tumors, which has implications for designing combination therapies. Importantly, they also provide an integrated dataset that combines in vitro and ex vivo studies with in vivo single-cell RNA-sequencing to characterize a novel BET inhibitor in GBM.

## Introduction

Despite intense research efforts, glioblastoma (GBM) remains an incurable disease^1^. Although enormous strides have been made in delineating the genetic and epigenetic changes that occur in GBM, patient survival at 5 years is still below 10%. Thus, there is an urgent need to identify more effective therapies for patients. A first step in this process is to identify and validate novel targets for therapeutic intervention.

We and others have demonstrated that epigenetic reader proteins with bromodomain and extra-terminal (BET) domains are promising therapeutic targets in GBM^2–10^. One such BET protein, bromodomain-containing protein 4 (BRD4), is a target in multiple cancers. In GBM tumors, inhibition or depletion of BRD4 reduces expression of oncogenes^11–13^. We and others have shown that small molecule BET inhibitors reduce the growth of GBM and other brain tumors by competing with the BET-histone interaction, thereby reducing transcription of oncogenes important for GBM cell proliferation^11,12,14,15^. Further, BRD4 inhibition may restore sensitivity to certain kinase inhibitors^10^ and BET inhibitors are well-tolerated in the clinic^16,17^.

Although multiple BET inhibitors have been developed, few are brain penetrant. The highly specific BET inhibitor JQ1 is widely used in the research setting and is indeed brain penetrant; however, it is not a clinical candidate due to chemical limitations such as a short half-life^10,18,19^. I-BET858 has a highly specific effect on transcriptional downregulation of genes controlled by brain derived neurotrophic factor (BDNF) causing an autism spectrum disorder phenotype in mice, however its effect on GBM cells has not been established^20^. MK-8628 has been used in a clinical study of 12 recurrent GBM patients (NCT02296476)^2^. However, the study was terminated when no increase in progression-free survival was observed^17^.

Based on a search of the literature and predicted physiochemical properties, we identified a potential brain penetrant BET inhibitor, which we modified to increase potency, generating UM-002. We demonstrate here that UM-002 is more potent than other BET inhibitors, including MK-8628 or JQ1, in reducing GBM cell proliferation in vitro. We also utilize UM-002 in a human brain organoid-based model of GBM and demonstrate that it effectively reduces proliferation and invasion ex vivo. Furthermore, we characterize the in vivo transcriptional response to UM-002 at single-cell resolution in an orthotopic mouse model of GBM and demonstrate that it reduces expression of cell cycle related genes in distinct cell populations. Collectively, these studies yield a novel BET inhibitor with brain penetrant properties that can be modified further for optimization for use in clinical trials as well as an integrated approach for characterizing novel brain penetrant BET inhibitors.

## Results

**Figure 1:**
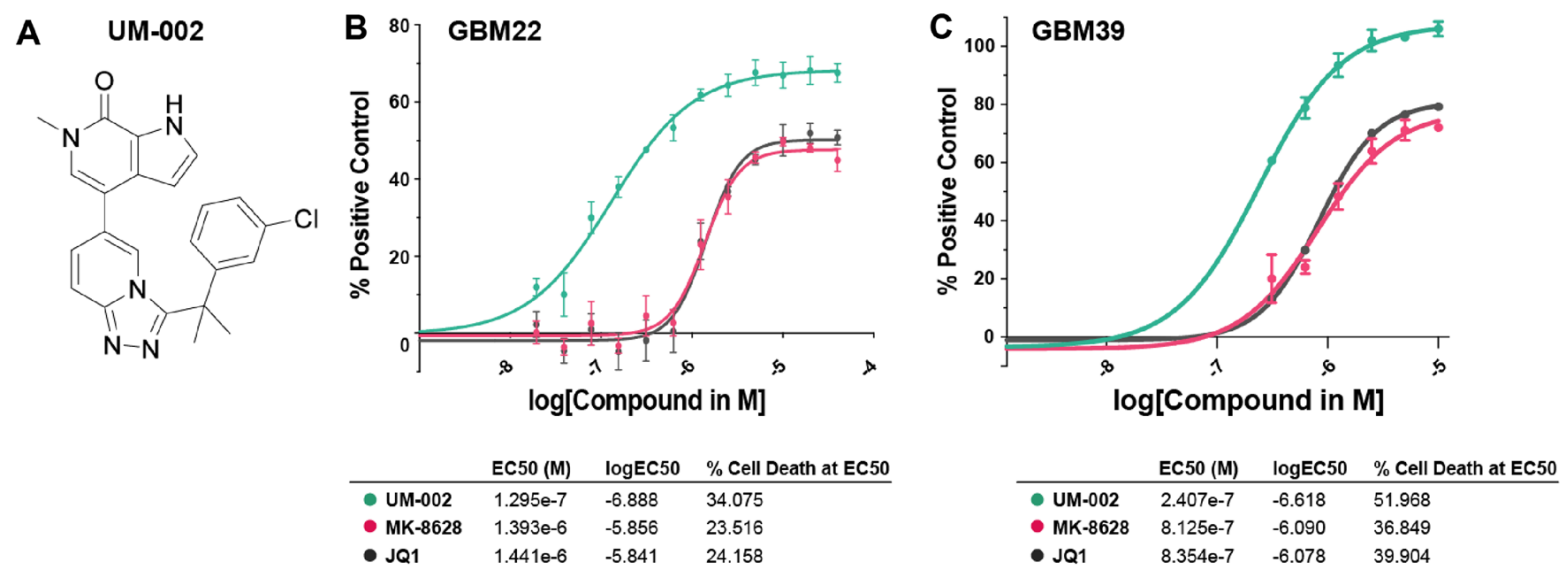
UM-002 reduces GBM cell proliferation in vitro. (**A**) Structure of UM-002. (**B,C**) UM-002 is more potent than JQ1 or MK-8628 in reducing GBM cell proliferation in vitro. GBM22 cells (**B**) or GBM39 cells (**C**) were treated with UM-002, MK-8628, or JQ1, starting at a dose of 10 µM, with 1:2 serial dilutions. Reduction in ATP levels was measured using CellTiter-Glo after 72 h of compound treatment and luminescence was normalized to the positive control. Dose response curves were calculated using a variable slope (4 parameters) fit. EC_50_ and the percentage of positive control at EC_50_ was calculated for each compound.

We and others previously demonstrated that BET bromodomain inhibition reduces GBM cell proliferation in vitro and in vivo using tool compounds^11–13^. However, although several hundred BET bromodomain protein inhibitors have been developed, few have been proven to be brain penetrant. As proper brain exposure is required to achieve full efficacy in reducing brain tumor expansion, we sought to generate brain penetrant BET inhibitors. To do this, we performed a literature search to identify all BET bromodomain inhibitors that may have proper physiochemical properties for brain penetrance and identified the triazolo[4,3-a]pyridin-6-yl)pyridinone chemotype (reference: WO2017/024,406-A1). Compounds derived from this scaffold have low molecular weights, low polar surface area values, and minimal NH moieties, all favoring brain penetration^21–23^. From this chemotype, we identified a previously developed BRD4 inhibitor, and we discovered that there is potential for increasing potency of this molecule while retaining properties of known brain penetrant compounds, yielding the novel BET inhibitor UM-002^24–26^ (Fig. 1A). UM-002 is highly potent BRD4 bromodomain inhibitor (EC_50_ values for BRD4-1 and BRD4-2 are 8.8 nM and 7.4 nM, respectively, Supplementary Figure S1A-B) and is brain penetrant (11% brain:plasma, Supplementary Figure S2). UM-002 is also more potent than JQ1 at inhibiting BRD2-1, BRD4-1, and BRD4-2 (Supplementary Table S1). UM-002 reduced GBM cell proliferation more than the commonly used BET inhibitor JQ1, or the brain penetrant BET inhibitor MK-8628 (Fig. 1B,C). Importantly, UM-002 did not affect the enzymatic activity of histone deacetylases, histone acetyltransferases (Supplementary Figure S1C), or potassium channels (Supplementary Figure S2). Collectively, these studies suggest that UM-002 is a novel selective brain penetrant BET inhibitor, that reduces GBM cell proliferation in vitro.

**Figure 2:**
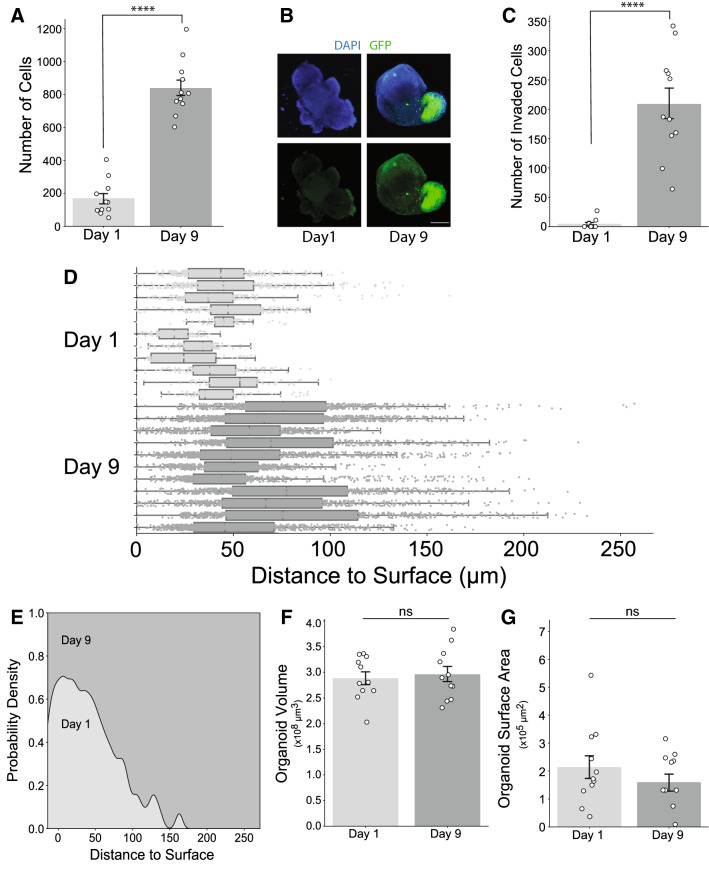
GBM cells proliferate and invade in a cerebral brain organoid-GBM co-culture model. (**A**) Total number of GBM cells at 1 and 9 days post co-culture; combined from three experimental replicates. (**B**) Representative maximum intensity projection images of fDISCO cleared cerebral brain organoid-GBM co-cultures. Top row: GFP-expressing GBM cells; bottom row: GFP merged with DAPI; scale bar for all images is in bottom right image and represents 300 µm. (**C**) Number of cells whose distance to the surface of the organoid exceeded the invasion threshold defined as the average third quartile value of the Day 9 group. (**D**) GBM cell distance to the nearest organoid surface. Boxplots represent individual organoids; data points are individual GBM cells. Distance was normalized by setting the minimum distance for each organoid to 0. (**E**) Normalized stacked kernel density estimation (KDE) of GBM cell distance to organoid surface. (**F**,**G**) Organoid volume (F) and surface area (G) measured using Imaris software. Statistical comparisons performed using an unpaired, two-tailed t test; **** ≤ 0.0001. Data from three independent biological replicates.

To validate these results in a system that more accurately recapitulates GBM in situ^27, 28^, we utilized an ex vivo, brain organoid-GBM co-culture model. In this model, induced pluripotent stem cells (iPSCs) are differentiated into mature human brain organoids and then co-cultured with GBM cells modified to express a fluorescent marker protein (GFP). Multiple variations of this and other organoid-GBM models have been developed recently^29^. Here we expanded the use of this particular organoid-GBM model by developing a method for quantifying both proliferation and invasion of GBM cells co-cultured with brain organoids. To quantify proliferation and invasion, we used the surface and spot modeling properties combined with the Shortest Distance Transformation features of Imaris (Fig. 2; detailed instructions provided in Experimental Procedures).

We first determined the time course of GBM cell proliferation and invasion after co-culture (Fig. 2). Organoid-GBM co-cultures were fixed at 1 and 9 days post co-culture for imaging. After 9 days, we observed that both the total number of GBM cells (Fig. 2A,B) and the number of invading cells (Fig. 2C–E) were significantly greater relative to the day 1 group. Additionally, we assessed the volume and surface area of the organoids in both groups and did not observe significant differences, suggesting that overall organoid viability is stable throughout co-culturing (Fig. 2F–G).

**Figure 3:**
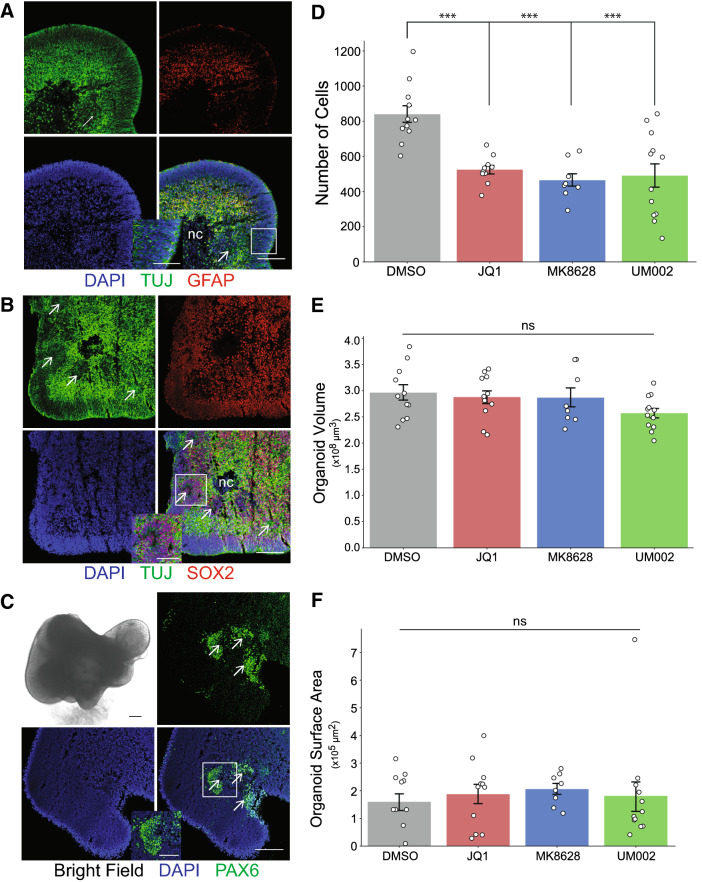
UM-002 reduces proliferation of GBM cells co-cultured with cerebral brain organoids. (**A–C**) Validation of cerebral brain organoid identity through immunohistochemical analysis of brain organoid cryosections (12 µm thickness) from 30-day-old organoids. White boxes are outlines of insets; arrows mark the center of typical cerebral brain organoid rosettes; nc = necrotic core. Bright field image in panel C is an uncleared, representative organoid. Scale bars in full panels represent 150 µm; scale bars in insets represent 75 µm. (**D**) Total number of GBM cells at 9 days post co-culture with cerebral brain organoids after treatment with DMSO, JQ1 (500 nM), MK8628 (1000 nM) or UM-002 (100 nM), with a final DMSO concentration of 0.05% across all treatment groups. Results are a combination of three independent experimental replicates from two organoid differentiation batches; brain organoids were matured in vitro for 96 or 98 days prior to co-culture with GBM cells. Data points represent the number of cells present in each organoid (y axis). (**E**,**F)** Organoid volume (E) and surface area (F) measured using Imaris software. Statistical comparisons performed using one-way ANOVA with Tukey HSD post-hoc; *** ≤ 0.001. Data shown are from three independent biological replicates.

**Figure 4:**
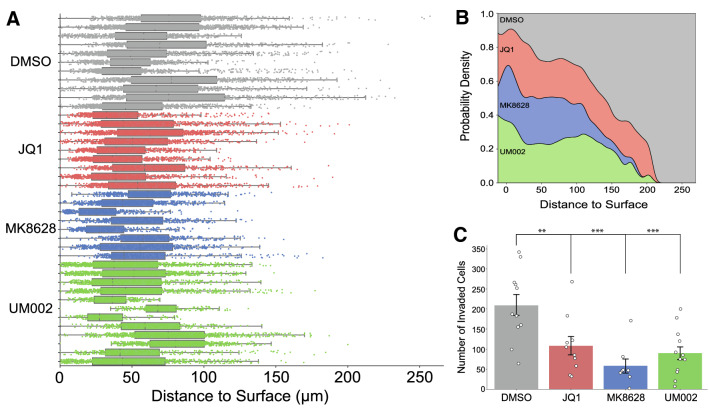
UM-002 reduces invasion of GBM cells into cerebral brain organoids. (**A**) GBM cell distance to the nearest organoid surface. Boxplots represent individual organoids; data points are individual GBM cells. Distance was normalized by setting the minimum distance for each organoid to 0. (**B**) Normalized stacked kernel density estimation (KDE) of GBM cell distance to organoid surface. (**C**) Number of GBM cells whose distance to the surface of the organoid exceeded the invasion threshold defined as the average third quartile value of the DMSO group. Statistical comparisons performed using a one-way ANOVA test with Tukey HSD post-hoc; ** ≤ 0.005; *** ≤ 0.001. Data shown are from three independent biological replicates.

To measure the effect of UM-002 on GBM cell proliferation and invasion in this model, we co-cultured GBM cells and cerebral brain organoids, then treated the co-cultures with DMSO, UM-002, JQ1 or MK-8628 for 9 days. All compounds reduced the total number of GBM cells relative to DMSO control (Fig. 3D) as well as the number of invading GBM cells (Fig. 4). Invasion of GBM cells was assessed by measuring the distance to surface for each individual GBM cell within the organoid (Fig. 4A). The number of GBM cells that invaded the internal space of the brain organoids was significantly less in the presence of UM-002 (Fig. 4B,C). Importantly, at the concentrations used, the compounds did not negatively impact brain organoid viability, as determined by organoid volume and surface area (Fig. 3E,F). These findings suggest that a therapeutic window can be achieved for limiting GBM tumor growth with minimal neurotoxicity from UM-002.

**Figure 5:**
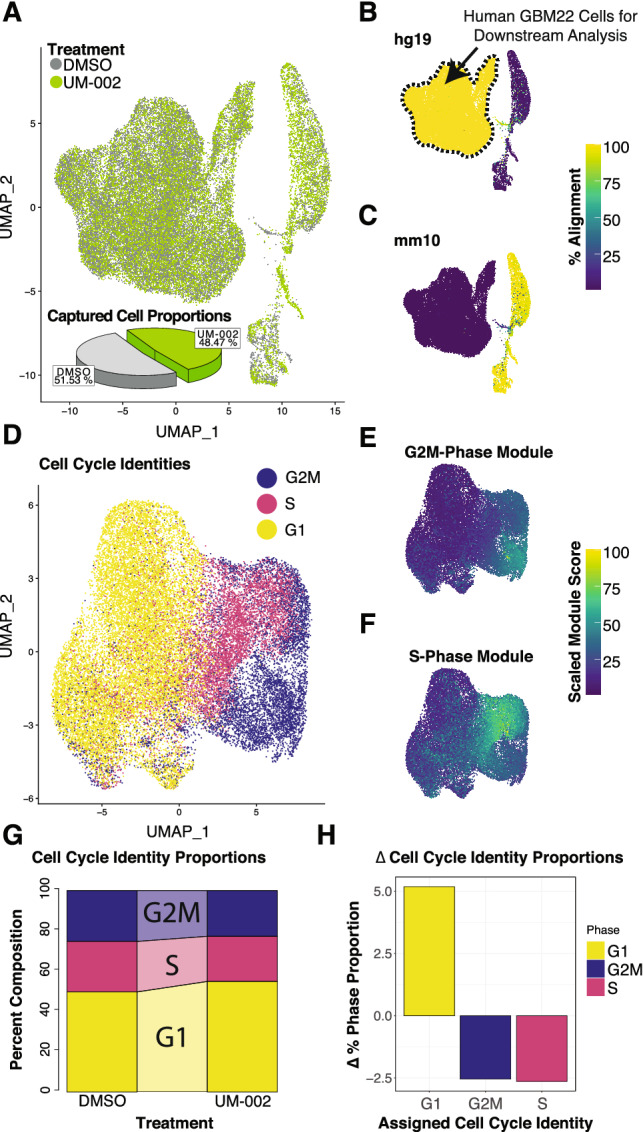
UM-002 induces changes in cell cycle related gene expression at single-cell resolution in vivo. (**A**) UMAP plot of the 38,200 total high-quality cell transcriptomes captured from both DMSO (gray) and UM-002 (green) treated PDX tumors. Cells are from tumors from 3 mice per treatment group. From DMSO treated mice, 18,452 cell transcriptomes were captured, while 19,748 were captured from UM-002 treated mice. (**B**,**C)** The GBM22 population of cells can be identified through analysis of individual cell percent alignment to hg19 (human) transcriptome (B), and percent alignment to mm10 (mouse) transcriptome. (C). (**D**) UMAP plot with pass-filter GBM22 cell transcriptomes (28,874 total GBM22, 14,878 DMSO treated, 13,996 UM-002 treated), colored by assigned cell cycle phase identity as determined using the R package Seurat^52–55,59^. (**E**) UMAP of GBM22 cell transcriptomes colored by G2M phase expression module score. (**F**) UMAP plot of GBM22 cell transcriptomes colored by S phase expression module score. (**G**) Connected bar plot showing the relative proportions of cells within G1, S, and G2M phases in DMSO and UM-002 treated tumors. (**H**) Bar plot showing changes in relative proportions (as percent) of cells within each phase of the cell cycle in UM-002 treated vs DMSO treated PDX tumors.

**Figure 6:**
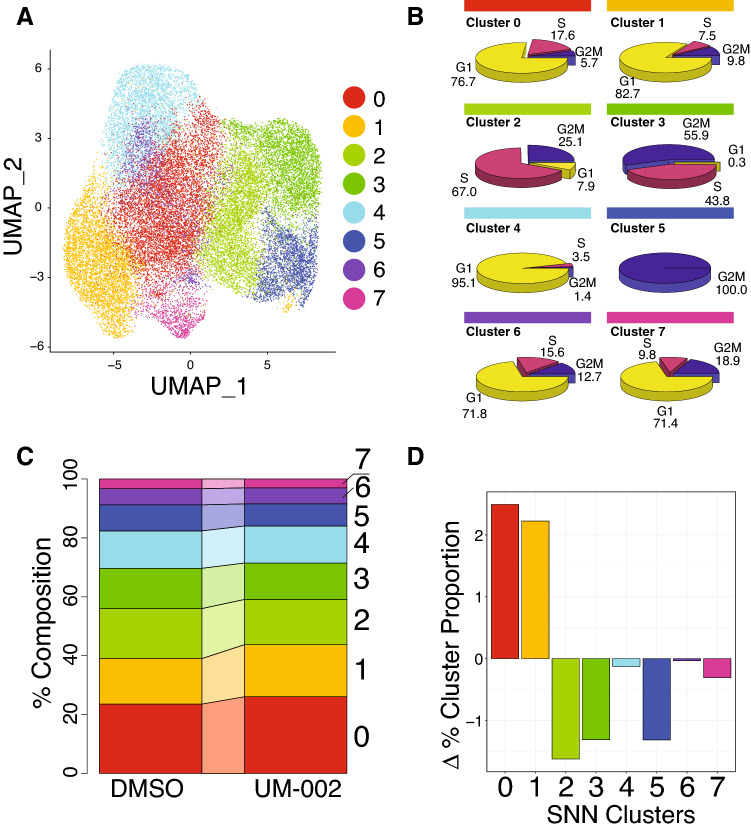
UM-002 treatment reduces the proportions of the most proliferative clusters of tumor cells in vivo. (**A**) UMAP of GBM22 cell transcriptomes captured from DMSO and UM-002-treated orthotopic xenografts, colored by unsupervised shared nearest neighbor (SNN) clustering. (**B**) Pie charts depicting relative proportions of cells within cell-cycle phases of each SNN cluster, annotations are percentages. (**C**) Connected bar plot showing the relative proportions of cells assigned to each SNN cluster within tumors treated with DMSO or UM-002. (**D**) Bar plot of change in relative percentage composition of each cluster in UM-002 treated tumors compared to DMSO treated control tumors.

**Figure 7:**
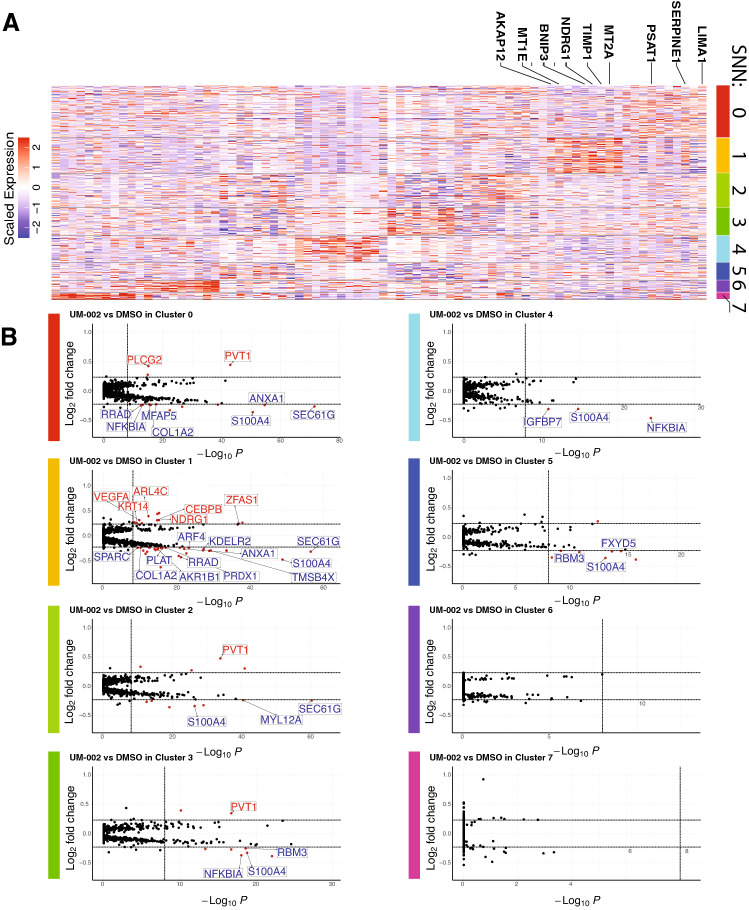
Single cell RNA-sequencing reveals distinct cell clusters with differentially expressed genes in response to UM-002 treatment in vivo. (**A**) Heat map of expression of top cluster defining transcripts, as determined by MAST^58^ and visualized using ggplot2 ^44,46,49^. Labeled genes are previously published GBM cell state markers^31^ found in clusters 0 and 1. (**B**) Volcano plot representation of MAST^58^ differential expression within clusters between UM-002 and DMSO- treated tumors. Selected transcripts are labeled. Full differential expression results can be found in Supplementary Data S1. Points in red are upregulated with log2FC > 0.23, Bonferroni corrected p-value < 10e-9. Points in blue are downregulated with 2 log2FC < -0.23, Bonferroni corrected p-value < 10e-9. Bonferroni corrections performed using all features in our dataset. Volcano plots generated using the R^43^ package EnhancedVolcano^50^.

To determine the effects of UM-002 in vivo, we treated GBM22 tumor-bearing mice with either DMSO (n = 3) or UM-002 (5 mg/kg/d) (n = 3) for 15 days and performed single-cell RNA-sequencing. Dual alignment of single-cell reads to both human (hg19) and mouse (mm10) transcriptomes allowed us to bioinformatically isolate human GBM22 PDX cells, and to demonstrate that UM-002 treatment increases the proportions of cells in G1 phase while decreasing the proportion of cells in S phase or G2M phase within this population (Fig. 5). Unsupervised SNN clustering revealed several proliferative cell clusters, including clusters 2 ,3 and 5, which were characterized by high proportions of cells determined to be within S or G2M phases (Fig. 6A,B). Additionally, these highly proliferative clusters are reduced in relative proportion by UM-002 treatment (Fig. 6C,D) and are characterized by the expression of transcripts such as *HIST1H4C* and *HIST1H1D*^30^, or other cell cycle markers such as *UHRF1* or *AURKA* (Fig. 7). Pathway analysis also revealed a significant enrichment in cell cycle related terms in these clusters, suggesting that cycling cells may be targeted by UM-002 (Supplementary Fig. 3). Clusters 0 and 1, which were observed to increase in relative proportion upon UM-002 treatment in vivo (Fig. 6D), are characterized by increased relative expression of mesenchymal GBM cell state markers such as *SERPINE1*, *MT2A*, *TIMP1*, *NDRG1*, *BNIP3*, and *MT1E*, as well as expression of the OPC-like markers *LIMA1* and *PSAT1* (Fig. 7A)^31^. Cluster 1 cells also showed a significant enrichment for pathways related to nonsense-mediated decay (NMD), which has been shown to regulate epithelial-to-mesenchymal transition (EMT) (Supplementary Fig. 3)^32^. Next, we performed differential expression testing within these identified clusters between tumors from UM-002 or DMSO treated mice (Fig. 7B). At least 19 differentially expressed transcripts have been previously implicated in GBM, including those implicated in EMT, invasion, proliferation, and treatment resistance (Supplementary Table S2). Collectively, these studies suggest that UM-002 affects the transcriptional landscape of GBM tumors in vivo by altering the proportion of cells in different cell cycle and invasive states.

## Discussion

Here we describe the development and characterization of a novel BET inhibitor UM-002 with brain penetrant properties that reduces GBM cell proliferation in vitro and ex vivo, and alters the proportion of cells within different cell cycle states in vivo*.* BET inhibition has been previously shown to reduce GBM cell proliferation in vitro and in vivo, however few BET inhibitors have been shown to be brain penetrant^11–13^. Our studies demonstrate that UM-002 is brain penetrant in naive mice and can affect GBM tumors in an orthotopic mouse model of GBM.

UM-002 is effective in reducing GBM proliferation within a human cerebral organoid system, a valuable new system for complementing findings from mouse models. Cerebral brain organoid-GBM models can be utilized to identify therapeutic doses for compounds. For example, we found that higher concentrations of UM-002 (> 500 nM) induced toxicity of the brain organoids themselves. This motivated us to utilize 100 nM, a concentration that was not toxic to the brain organoids but remained effective in reducing GBM cell proliferation. Interestingly, this concentration also reduced invasion of GBM cells into cerebral organoids, although we cannot exclude the possibility that invasion inhibition via UM-002 is not merely a consequence of reducing proliferation.

In addition to our findings on reducing GBM cell proliferation, our in vivo studies suggest that UM-002 is reaching GBM tumors in the brain. We observed a marked decrease of cells in G2M and S phases, and an increase of cells in G1 phase in tumors from mice treated with UM-002 (Fig. 5D–F). This is consistent with previous reports demonstrating that BET inhibition induces cell cycle arrest in the G1 phase in multiple cancers including GBM^6,8,33,34^. These findings are also consistent with our results from the organoid-GBM model showing that BET inhibition reduces GBM proliferation. In vivo, UM-002 treated tumors had a shift in cell populations favoring a significant decrease in cells within proliferative states, and an increase in cells within non-proliferative states—marked by the expression of genes related to GBM cell mesenchymal states, such as *SERPINE1* and *MT1E*^31^ (Fig. 7A). Collectively, these studies indicate that UM-002 affects GBM tumors in vivo by reducing the proportion of proliferating cells and decreasing expression of cell cycle genes.

Moreover, the clusters of cells with proportions significantly increased by UM-002 treatment showed an enrichment in pathways that could be targeted by other compounds that may synergize with BET inhibitors. For example, pathway analysis demonstrated that cells from UM-002 treated tumors were enriched in pathways related to nonsense-mediated decay (NMD) (Supplementary Fig. 3), which may provide a justification for combination treatment with an NMD inhibitor. Therefore, it will be crucial to further characterize cellular survival pathways following BET inhibitor treatment at the single-cell level to design effective combination treatments with UM-002 or other brain penetrant BET inhibitors.

To our knowledge, this is the first report that BET inhibition differentially affects sub-populations in GBM in vivo. At present, it is unclear if the shifts in cell populations are due to UM-002 selectively targeting transcriptionally distinct populations or if BET inhibition may alter GBM cellular transcriptional states. Phenotypic and transcriptional cell state transitions have been shown to be non-hierarchical and reversible in GBM and may be occurring by a mechanism of exploratory adaptation to the tumor microenvironment^35,36^. Future studies are required to determine whether BET inhibition yields resistant cells that utilize these adaptation mechanisms in GBM clinical trials.

## Methods

### General synthetic procedures

**Scheme 1:**
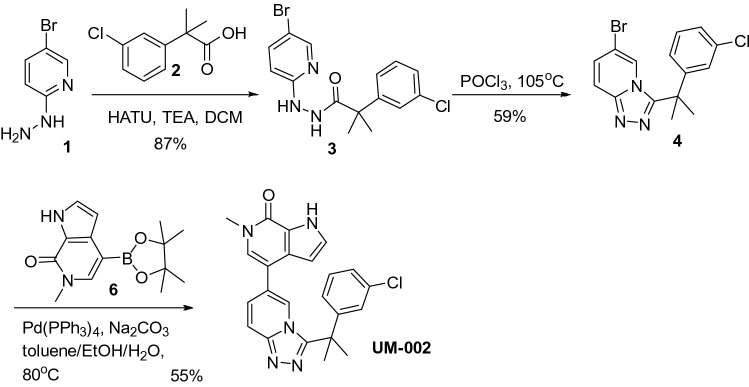
Synthesis of compound UM-002.

All starting materials, solvents, catalysts, and general chemicals are from commercial sources and were used without further purification unless otherwise indicated (Scheme 1). ^1^H-NMR spectra were recorded on Bruker Avance III 400 MHz and Varian Mercury Plus 400 MHz and TMS was used as an internal standard. LC/MS was performed on a quadrupole Mass Spectrometer on Agilent LC/MSD 1200 Series (Column: BP-C18 (50 × 4.6 mm, 5 μm) operating in ES ( +) or (−) ionization mode; T = 30 °C; flow rate = 1.5 mL/min; detected wavelength: 254 nm.

### Synthesis of compound 3

(N'-(5-bromopyridin-2-yl)-2-(3-chlorophenyl)-2-methylpropanehydrazide). HATU (2-(3H-^33^triazolo[4,5-b]pyridin-3-yl)-1,1,3,3-tetramethylisouronium hexafluorophosphate(V)) was added to a suspension of Compound **1**(1.0 equiv.), compound **2** (1.1 equiv.), and TEA (triethyl amine, 4 equiv.) in DCM. The solution was stirred at room temperature for 2 h (LC–MS showed that starting material compound **1** was completely consumed). The solvents were removed under vacuum, and the residue was suspended in ethyl acetate, washed by brine (2x), 1 N citric acid (1x), brine (1x), saturated Na_2_CO_3_ (2x), and brine (2x), and dried over anhydrous Na_2_SO_4_. The solvents were removed under vacuum, and the residue was subjected to flash chromatograph using MeOH/DCM to give compound **3**.

### Synthesis of compound 4

(6-bromo-3-(2-(3-chlorophenyl)propan-2-yl)-[1,2,4]triazolo[4,3-a]pyridine). Compound **3** obtained above was suspended in POCl_3_. The suspension was heated to and stirred at 105 °C for 3 h. After removing the remaining POCl_3_ by Rotovapor, the residue was cooled to room temperature, suspended in ethyl acetate, washed by brine (2x), saturated Na_2_CO_3_ (2x), and brine (2x), and dried over anhydrous Na_2_SO_4_. The solvents were then removed under vacuum to give compound **4**, which was used directly in the next step without further purification.

### Synthesis of UM-002

(5-(3-(2-(3-chlorophenyl)propan-2-yl)-[1,2,4]triazolo[4,3-a]pyridin-6-yl)-1,3-dimethylpyridin-2(1H)-one). House vacuum was applied to a stirred suspension of Compound **4** (1 equiv.)**,** Na_2_CO_3_ (5 equiv.), and Compound **5** in Toluene/Ethanol/Water (6:3:1 by volume) to remove air for 30 min. Pd(PPh3)4 (10%) was added to this suspension quickly, the pressure flask was sealed, heated to, and stirred at 80 °C overnight. After removing the solvents by Rotovapor, the residue was suspended in ethyl acetate, washed by brine (2x), 1 N HCl (2x), brine (1x), saturated Na_2_CO_3_ (2x), and brine (2x), and dried over anhydrous Na_2_SO_4_. The solvents were then removed under vacuum to give the crude product, which was then subjected to preparative HPLC to give the final product compound **UM-002**. Chemical Formula: C_23_H_20_ClN_5_O, LC–MS, calculated (M + H): 418, obtained (M + H)^+^: 418. ^1^H-NMR (400 MHz, DMSO-d6), δ (ppm) 12.21 (s, 1H), 7.90 (d, J = 10.4 Hz, 1H), 7.58 (d, J = 8.8 Hz, 2H), 7.48 (d, J = 8.4 Hz, 1H), 7.41 (t, J = 8.0 Hz, 3H), 7.23 (t, J = 2.8 Hz, 1H), 7.04 (d, J = 8.0 Hz, 1H), 5.31 (t, J = 2.2 Hz, 1H), 3.54 (s, 3H), 1.86 (s, 6H).

### Characterization of novel BET inhibitors:

#### Metabolic stability assay

UM-002 was tested in human and mouse hepatic microsomes at a concentration of 1 mg/ml as previously described^37^.

#### Plasma protein binding

Plasma protein binding was determined using equilibrium dialysis with 5 µM drug in plasma.

#### P450 inhibition

Cytochrome P450 inhibition was assessed with or without UM-002 in human liver microsomes using a panel of marker substrates, as previously described^37^.

#### Brain penetrance assays

UM-002 was tested in seven to eight-week-old male C57Bl/6 mice. UM-002 was dosed at 10 mg/kg IP dose using a formulation of 10% DMSO, 15% tween80 and 75% water. Test compounds were first dissolved in DMSO prior to the addition of additional organic excipients and finally water or saline. Final formulation concentration was 1 mg/ml. Two hours post administration, blood and brain were obtained from mice (n = 3). Plasma was obtained by centrifugation and immediately frozen. Compound levels were determined by mass spectrometry using an ABSciex 5500 mass spectrometer using multiple reaction monitoring by comparison against separate standard curves prepared in blank mouse plasma and blank mouse brain homogenate. All work was conducted in the Scripps Florida vivarium, which is fully AAALAC accredited. Procedures were approved by the Scripps Florida IACUC, protocol number 15–022. All procedures were performed in accordance with guidelines and regulations, including ARRIVE guidelines.

#### Potassium channel blockage

The effect of UM-002 on cardiac function was tested using an Ether-a-go-go-related gene potassium channel 1 (hERG) binding assay performed at Reaction Biology Corporation. Percentage channel blockage was calculated compared to a DMSO control.

#### BET protein activity assays

BET inhibition assays were performed at Reaction Biology Corporation as previously described^38^. The disruption of BET protein binding to pre-acetylated biotin-histone 4 peptides was measured using a biochemical AlphaScreen assay, which detects proximity of the conjugated donor and acceptor beads using transient singlet oxygen.

#### HDAC profiling

HDAC reference compounds Trichostatin A and TMP269 were tested in a 10-dose IC50 with threefold serial dilution, starting at 10 µM or 1 µM, respectively. IC50 values were calculated using GraphPad PRISM (version 8.4.3) 4 curve fitting with sigmoidal dose–response. Percentage of enzyme activity relative to DMSO control was calculated for UM-002 at a concentration of 500 nM.

#### HAT profiling

UM-002 was tested against a panel of seven histone acetyltransferases at a dose of 500 nM. Control compounds C646 and Anacardic Acid were tested in 10-dose IC50 with threefold serial dilutions starting at 100 µM. Percentage of enzyme activity relative to DMSO control was calculated, such that the DMSO no-inhibitor control was 100% activity.

### GBM cell maintenance and proliferation assays:

#### GBM PDX cell culture

The patient derived xenograft (PDX) glioblastoma cell lines, GBM22 and GBM39, were obtained from the Mayo Clinic Brain Tumor PDX national resource. Cells were cultured as previously described^39,40^. Since genetic drift is known to occur with passage in vivo and in vitro, a stock of early passage tumor tissue is maintained in storage and xenograft lines are passaged a maximum of 20 times before restoration from early passage tissue. For in vitro studies, cells were maintained at an early passage, for a maximum of 30 days in culture, or approximately 5 passages. Cells were cultured in Dulbecco’s Modified Eagle’s medium (DMEM):F12 with 10% fetal bovine serum and 1% penicillin and streptomycin (pen/strep).

#### In vitro dose response compound screens

PDX cells were plated at 3000 cells/well in a 384 well flat bottom plate, and adherent cultures were established overnight. Cells were then treated with UM-002, MK-8628, or JQ1 in ten 1:2 serial dilutions, starting at 10 µM with a final DMSO concentration of 0.1%. Following a 72-h incubation, CellTiter-Glo (Promega, Wisconsin, USA) was used to measure ATP content and luminescence was read on an EnVision plate reader, as previously described^39^. Luminescence was normalized to 10 µM Velcade as a positive control, and 0.1% DMSO as a negative control. Dose response curves were calculated in GraphPad PRISM (version 8.4.3) using a variable slope (four parameter) fit.

### Brain organoid model for glioblastoma tumor treatment and imaging

#### Brain organoid production, co-culturing and treatment

On day 0 of organoid production, induced pluripotent stem cells (iPSCs), at ~ 80% confluence, were dissociated into small clumps with gentle cell dissociation reagent (StemCell Technologies, 07174) and plated into round-bottom, low-attachment 96 well plates (ThermoFisher, 174925; ~ 15,000 cells per well). Initial media, consisting of mTeSR Plus (Stem Cell Technologies, 05825) supplemented with 5 µm A-83 (Peprotech, 9094360) and 5 µm Dorsomorphin (Peprotech, 8666430), was replaced every other day starting at day 2 by removing half of the media in each well (100 µL) and replacing with fresh media three times. On day 8, media was changed to a base media of Neurobasal A media (Gibco, 10888022) with NeuroCult SM1 (StemCell Technologies, 05711), glutamax (Gibco, 35050061), and Pen/strep (Gibco, 15140122) supplemented with EGF (20 ng/ml; Alomone Labs, E-100) and FGF2 (20 ng/ml; Alomone Labs, F-170). Media was similarly exchanged every other day until day 20 when the mitogenic supplements were exchanged for differentiating factors: BDNF (20 ng/ml; Alomone Labs, B-250) and NT3 (20 ng/ml; Alomone Labs, N-260) in the same base media. After two weeks in differentiation media, organoids could be maintained in the base media for > 100 days^41^.

#### Organoid sectioning and staining

Organoids were fixed with 4% paraformaldehyde for 2 h at room temperature (RT) with gentle rotation on an orbital shaker. Fixed organoids were washed 3 × with PBS and stored at 4  °C. Before sectioning, to cryoprotect the tissue, fixed organoids were left in a 30% sucrose solution (in PBS) overnight. Cryoprotected organoids were placed in plastic molds with O.C.T. compound (optimal cutting temperature; Fisher Scientific, 23–730-571) for 30 min at RT before freezing with liquid nitrogen. Frozen tissue blocks were placed in the cryostat at –22 °C and left to equilibrate for 30 min before cutting in 12 µm sections. Sections were transferred to glass microscope slides (Fisher Scientific, 22–037-246) and stored in an air-tight bag at 4 °C until staining. Before staining, slides were dried on a hot plate at 80 °C for 3 min and the OCT residue carefully removed with fine forceps. Slides were washed 3 × with PBS then simultaneously permeabilized and blocked in antibody buffer with 0.2% Triton X and 20% goat serum. After washing again 3 × with PBS, sections were incubated with primary antibodies diluted in antibody buffer with 10% goat serum overnight at 4 °C in a dark, humified chamber. Primary antibodies and dilutions were as follows: beta III tubulin, 1:500 (TUJ; Abcam, ab78078); glial fibrillary acidic protein, 1:200 (GFAP; Agilent, GA524); SRY-Box Transcription Factor 2, 1:400 (SOX2; Abcam, ab97959); paired box 6, 1:20 (PAX6; Novus Biologicals, NBP2-34705AF488). The following sections were washed 3 × in PBS and incubated with secondary antibodies diluted (1:500) in antibody buffer with 10% goat serum for 2 h at RT in a dark, humidified chamber. Following incubation of secondary antibodies, sections were washed 3×, incubated with DAPI for 15 min, washed 3 × again and mounted with Fluoroshield (Abcam, ab104135). Stained sections were imaged with a Zeiss LSM 710 confocal microscope with a 20 × objective, an 8-bit or 16-bit imaging depth and a 1024 × 1024 imaging frame; pixel intensity values were averaged 12 times (Fig. 3A–C).

#### Co-culturing and treatment

GBM22 cells were dissociated into a single cell suspension with Trypsin (Gibco, 15,400,054) and seeded (17,500 cells/well) into round-bottom, low-attachment 96-well plates containing a single mature organoid per well. Cells were seeded by removing 100 µL of media and replacing with 100 µL of cell suspension. GBM cells were allowed to settle around the organoid and attach overnight. After 24 h (day 1 of co-culturing) organoids were either fixed (see below) and stored in PBS as an untreated control or treated with compounds. For treatment, compounds were dissolved in DMSO and diluted in organoid maintenance media. Organoids were dosed with 500 nM JQ1, 1000 nM MK-8628, 100 nM UM-002, or DMSO, at a final DMSO concentration 0.05%. On the first day of treatment, organoids were treated by removing half of the media (100 µL) and replacing with 100 µL of a 2 × compound solution. Compounds were renewed with 1 × solutions in the new media every other day following the normal media replacement regimen until the conclusion of the experiment. To ensure unbiased results, compound treatments and subsequent analyses were performed under experimental blinding.

#### Organoid clearing and imaging

At the conclusion of treatment (or on day 1 for the untreated group), organoid-GBM co-cultures were carefully transferred from their 96-well plates to small glass jars. Organoids were fixed with 4% paraformaldehyde for 2 h at RT with gentle rotation on an orbital shaker. Fixed organoids were washed 3 × and stored in PBS at 4 °C. Organoids were cleared according to the organic solvent-based clearing protocol: FDISCO^42^. Briefly, organoids were dehydrated with a series of incubations with increasing concentrations of tetrahydrofuran (50%–60 min, 70%–30 min, 80%–30 min, 100%–30 min × 2; Sigma, 186,562-12X100ML). Organoids were left overnight in 100% THF; and the following morning, ~ 30 min prior to imaging, the solution was replaced with dibenzyl ether (DBE; Sigma 108,014-1 KG) to complete the clearing process by refractive index matching. All clearing incubations were performed in the dark at 4 °C with gentle rocking. Cleared organoids were imaged on a Zeiss LSM 710 confocal microscope with a 10× objective, 8-bit imaging depth and a 1024 × 1024 imaging frame; pixel intensity values were averaged 4 times. To maintain uniformity between organoids, exactly 85 optical sections were imaged for each organoid with a z-step of 3 µm for a total z-distance of 255 µm. fDISCO cleared tissues (including brain organoids) uniformly shrink during dehydration, thus a 255 µm z-stack was sufficient to capture a majority of the organoid.

#### Image processing and data analysis

Z-stacks were processed using Imaris Software (version 9.6.0; https://imaris.oxinst.com/versions/9-6) because Imaris surfaces can be used to contour the surface of objects and Imaris spots can be used to locate cells in 3-dimensional space. The bottom of the organoid began at or close to the first optical section of the z-stack so in order to accurately model the surface, 50 black (blank) slices were added to the bottom of the image (Edit > Add Slices). Then, an Imaris surface was created using the DAPI channel (Surface Detail was set to 25 µm). This surface correctly modeled the surface of the organoid with the exception of the top of the z-stack where the surface was enclosed. To prevent this, a new channel was created with a mask of everything outside the original surface (with original surface selected, under Edit tab of Surface Properties: Mask Selection. Set voxels outside to 150; duplicate surface to a new channel). A second surface was then generated from this new channel (Surface Detail again set to 25 µm) which correctly modeled the organoid surface. GBM cells were located by creating Imaris spots (size parameter set to 10 µm diameter). Thus, when the Shortest Distance Transformation option was enabled for both the second surface and spots, each GBM cell’s distance to the nearest surface of the organoid was generated. All data for surfaces and spots was exported to CSV files for analysis. Data was extracted and analyzed with a Python script available at https://github.com/rybinmj/gbm_org_analysis. Statistical comparisons performed using an unpaired, two-tailed t test or one-way ANOVA with Tukey HSD post-hoc.

#### In vivo orthotopic intracranial glioblastoma tumor treatment and sequencing

### Intracranial GBM PDX tumor implantation

GBM22 cells were transduced with lentiviral libraries expressing GFP. Cells were visualized to confirm GFP expression prior to intracranial implantation into mice. Cells were implanted in mice using a previously described protocol^40^. Briefly, Nu/Nu mice (Charles River Laboratory) were anesthetized with ketamine (100 mg/kg) and xylazine (10 mg/kg), and a hole was drilled through the skull (1 mm anterior and 2 mm lateral to bregma). Using a Hamilton syringe 150,000 GBM22 cells suspended in PBS were injected at a depth of 2 mm. Skin flaps were sealed using surgical glue or sutures and mice were given buprenorphine as analgesic. Procedures were approved by the University of Miami IACUC, protocol number 18–014. All procedures were performed in accordance with guidelines and regulations, including ARRIVE guidelines.

#### GBM tumor treatment with UM-002 or DMSO

Following tumor implantation, mice recovered for a period of 6 days, followed by treatment with UM-002 (5 mg/kg/day) or DMSO. Drug formulation was as follows: 10% DMSO, 40% PEG400, 50% PBS. Mice were injected intraperitoneally 5 days per week for 3 weeks and monitored daily for signs of cognitive decline. Cells from tumors from three individual mice within each treatment group were isolated for single cell RNA-sequencing.

#### Single cell sequencing analysis

Tumor-bearing mice were perfused with PBS, and their brains were removed. Tumor was isolated and dissociated using Papain (Worthington Biochemical). Cells were washed two times using ice-cold PBS with 0.1% BSA (w/v), checked for viability using AO/PI dye and a Nexcelom K2 Cellometer, and were loaded into a 10X Chromium chip using 10X Genomics NextGEM 3’ chemistry targeting the capture of approximately 10,000 cells per sample. With each individual animal experiment, paired-end library sequencing of DMSO and UM-002 treated single-cell libraries was performed in tandem on a single S1 NovaSeq flow cell, with asymmetric read lengths as recommended by 10X Genomics. Base-calls were input into CellRanger for demultiplexing and fastq generation. Alignment and gene quantification were first performed in tandem to a dual-species reference transcriptome for both hg19 (human) and mm10 (mouse), obtained from 10X Genomics. (refdata-cellranger-hg19-and-mm10-3.0.0.). Using the dual-transcriptome alignment, 18,452 total cells were captured from 3 DMSO treated tumors, while 19,748 total cells were captured from 3 UM-002 treated tumors. Further analysis and figure generation was performed in R^43–51^. Using the R package Seurat (v. 4.0.1)^52–55^, the percentage of raw UMI counts within each cell aligning to either mm10 or hg19 transcriptomes was calculated and used in tandem with SNN clustering to separate out human GBM22 cells from contaminating mouse cells (Fig. 5A–C). GBM22 cell barcodes were then used to isolate these human cells from the data aligned to the GrCH38 (human) transcriptome for downstream gene expression analysis. Outlier cells were removed based on UMI count, detected gene count, and percent of mitochondrial transcript counts. Cells with less than 200 or more than 6000 detected genes, or with more than 10% of mitochondrial genome alignment were discarded. Following filtering, a total of 28,874 high-quality GBM22 cell transcriptomes were captured, comprised of 14,878 GBM22 cell transcriptomes from DMSO treated tumors, and 13,996 GBM22 cell transcriptomes from UM-002 treated tumors (Fig. 5D). For visualization, DMSO and UM-002 datasets were first individually transformed using a regularized negative binomial regression (scTransform)^56^. Pearson residuals were then used to identify anchor features and to integrate the individual datasets for dimensionality reduction and shared-nearest-neighbor (SNN) clustering^53^. An ideal clustering resolution was determined using the R package Clustree (v. 0.4.3)^57^. Differential expression was performed using MAST (Model-based analysis of single-cell transcriptomics)^58^ using log-transformed counts to identify cluster markers and to identify differentially expressed genes across treatment arms. Cell-cycle analysis was performed using Seurat. Cells were scored for their expression of either a G2M or S phase signature using a technique outlined by Tirosh et al. (2016)^59^. Based on these scores, cells were binned based on their likely cell cycle status. Cells with lower G2M and S scores were assigned to G1. These scores and proportions were compared across treatment arms (Fig. 5D–H), and between SNN clusters to characterize distinct populations of cells. Heat map of cluster markers and differentially expressed genes was generated using the R package ggplot2^44,46,49^ (Fig. 7A). Volcano plots of differentially expressed genes between treatment arms were generated using the R package EnhancedVolcano^50^ (Fig. 7B). Analysis pipeline is available at https://github.com/AyadLab/UM002-scPerturbation.

## Supplementary Information

Supplementary Information 1.

Supplementary Information 2.

## Data Availability

All data will be made publicly available prior to acceptance at GEO accession GSE173146. Analysis pipeline for human cerebral brain organoid proliferation and invasion measurements is available at https://github.com/rybinmj/GbmInvasion. Analysis pipeline for scRNA-seq is available at https://github.com/AyadLab/UM002-scPerturbation.
