# Supplementary material for: The novel BET inhibitor UM-002 reduces glioblastoma cell proliferation and invasion
Source: Sci Rep. 2021 Dec 3;11:23370. doi: 10.1038/s41598-021-02584-6 (PMC8642539; doi:10.1038/s41598-021-02584-6)
Supplement: Supplementary file 1 — Supplementary Information 1. [file 41598_2021_2584_MOESM1_ESM.docx]

**SUPPLEMENTARY INFORMATION**

Title: The novel BET inhibitor UM-002 reduces glioblastoma cell proliferation and invasion

Anna M. Jermakowicz, Matthew J. Rybin, Robert K. Suter, Jann N. Sarkaria, Zane Zeier, Yangbo Feng, and Nagi G. Ayad

**Supplementary Figure S1**: UM-002 does not inhibit HDACs and HATs……..…………..…….S-2

**Supplementary Figure S2**: UM-002 is brain penetrant……………………………...……………S-3

**Supplementary Figure S3**: Pathway and gene ontology enrichment characterizes

distinct GBM22 clusters based on invasion and cell cycle state…….…..…………..S-4

**Supplementary Table S1**: UM-002 inhibits BET bromodomain proteins ………………………S-6

**Supplementary Table S2**: UM-002 altered the expression of GBM relevant genes *in vivo*….S-7

**Supplementary Data S1**: MAST differential expression within clusters between UM-002

and DMSO treated tumors reveals transcriptionally distinct features


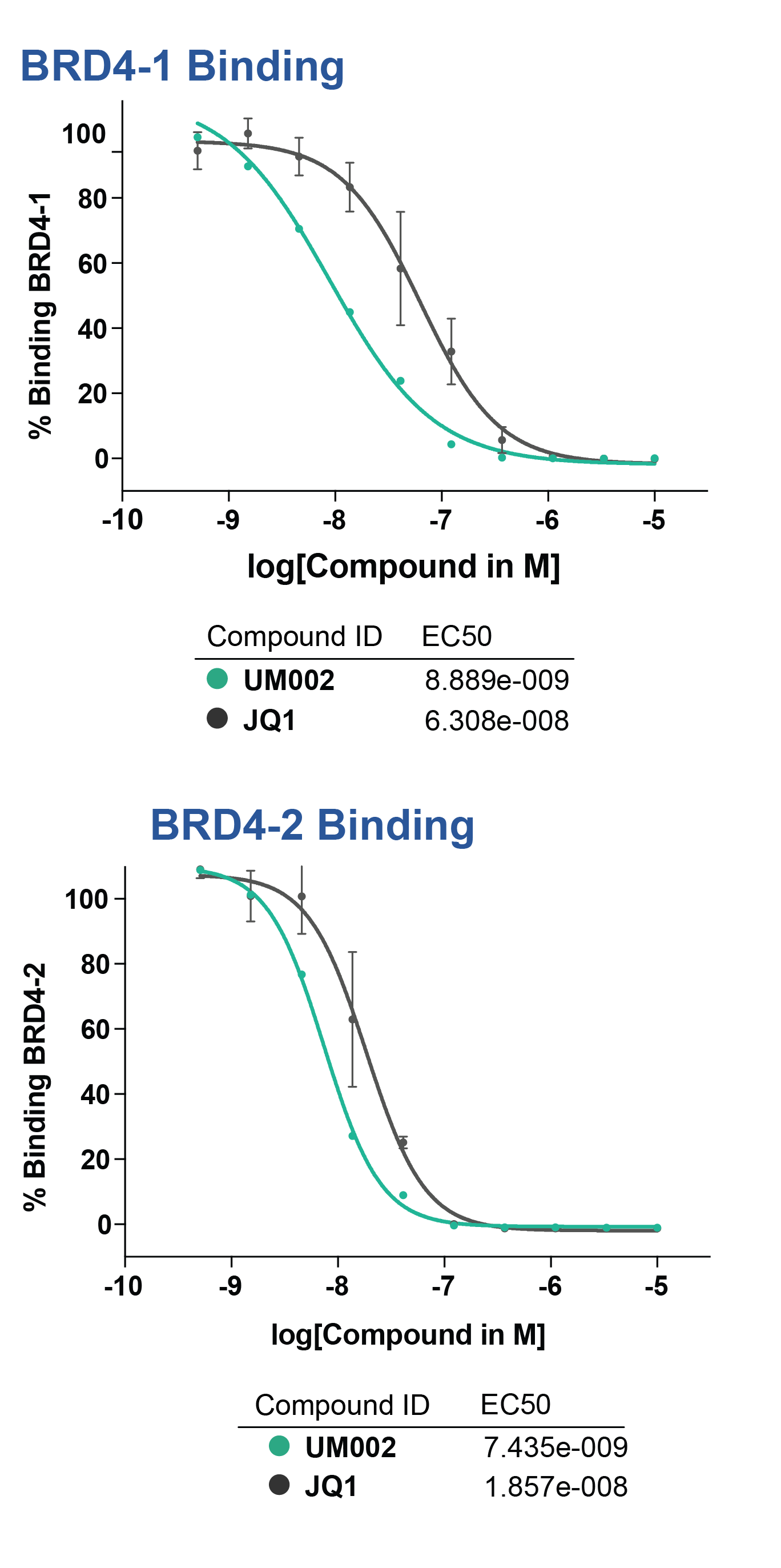

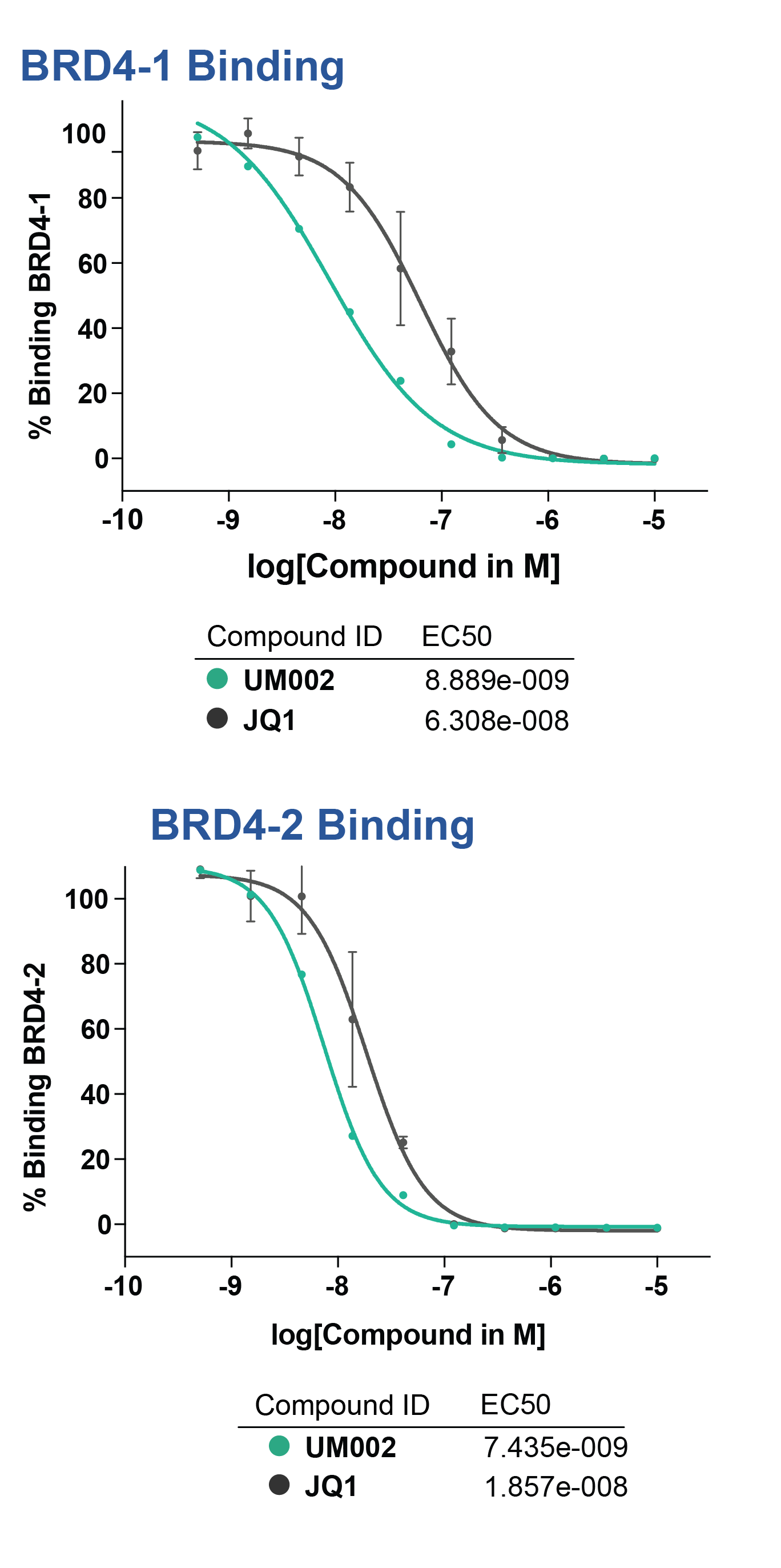

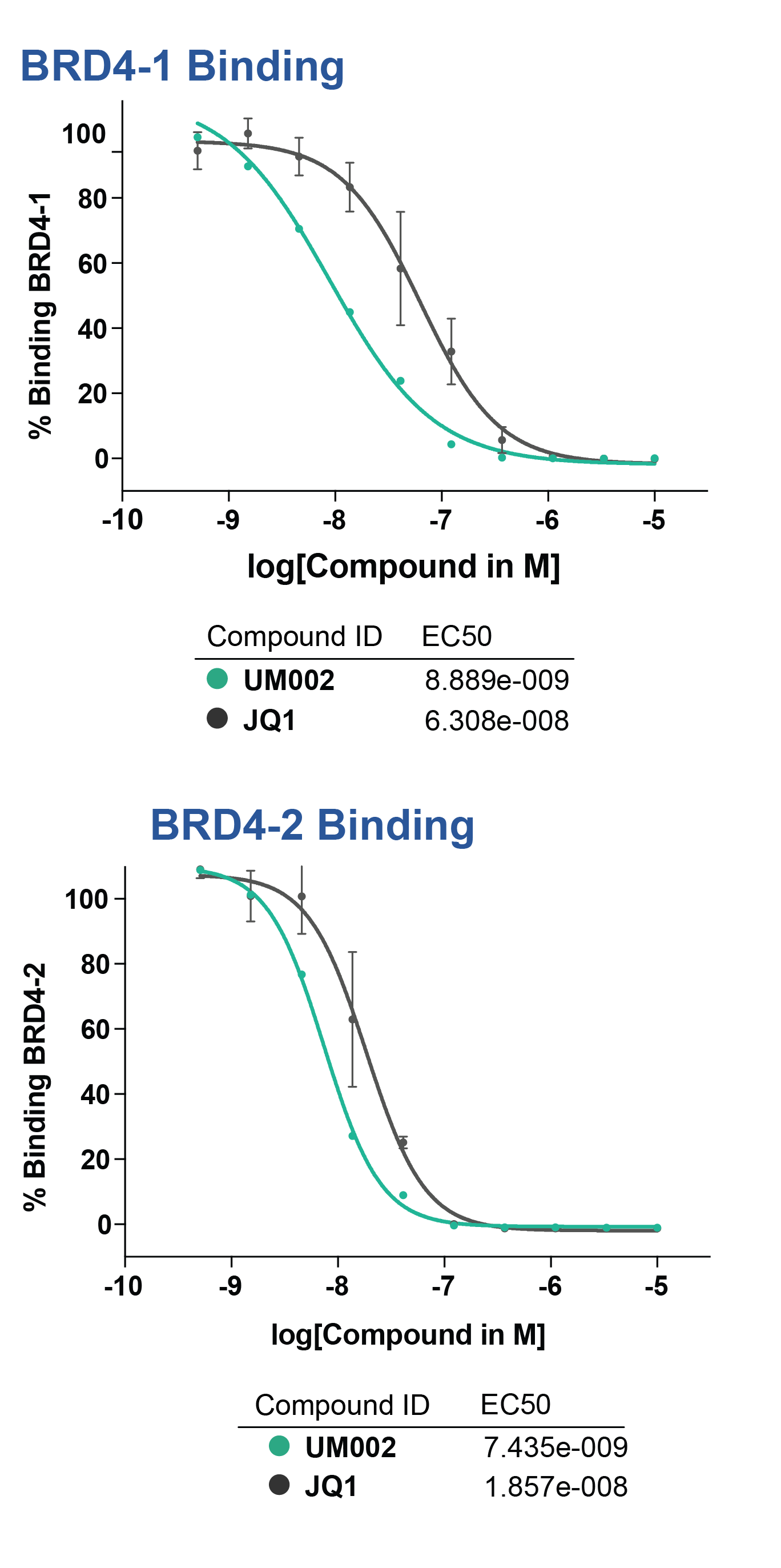

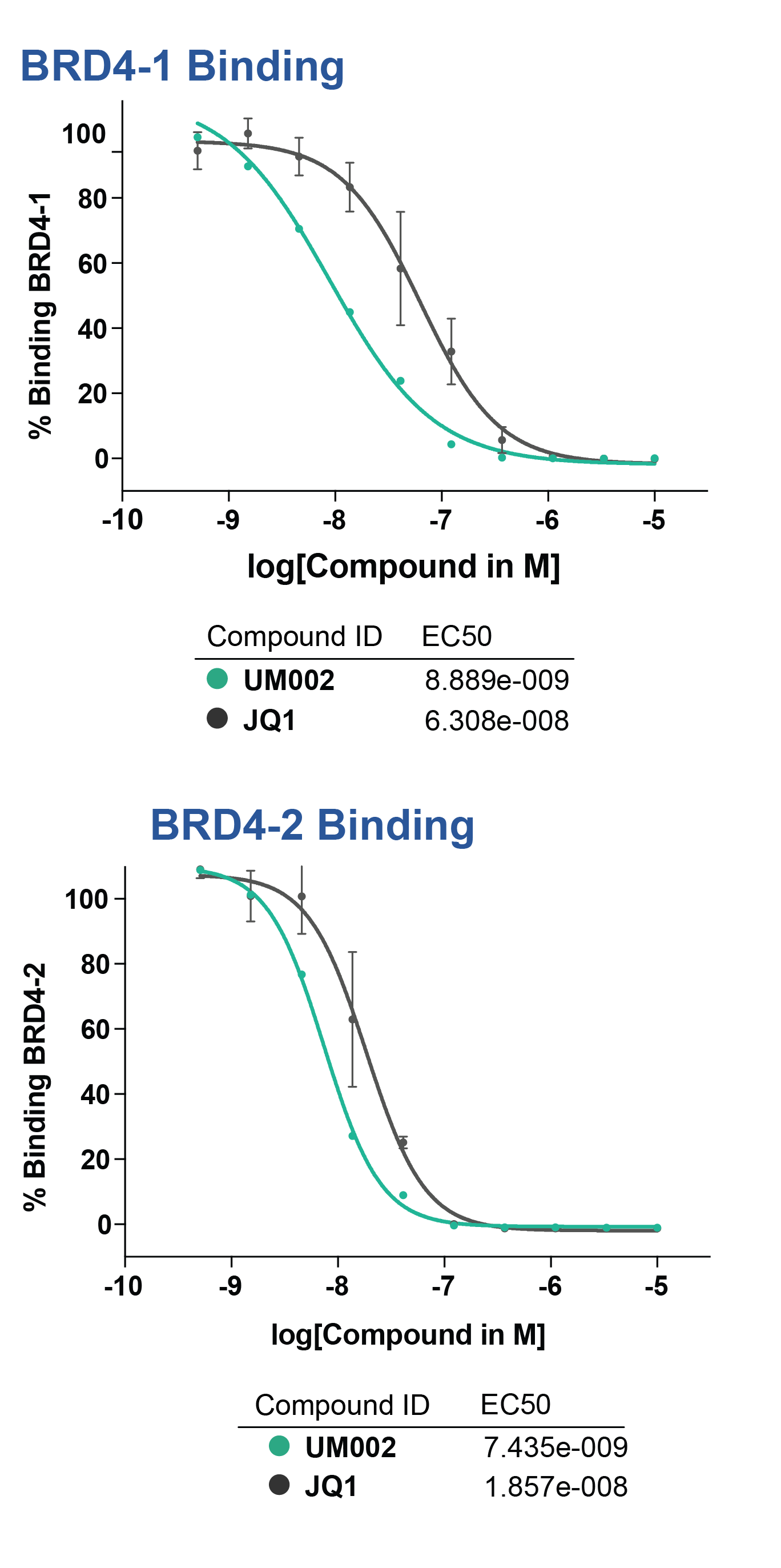

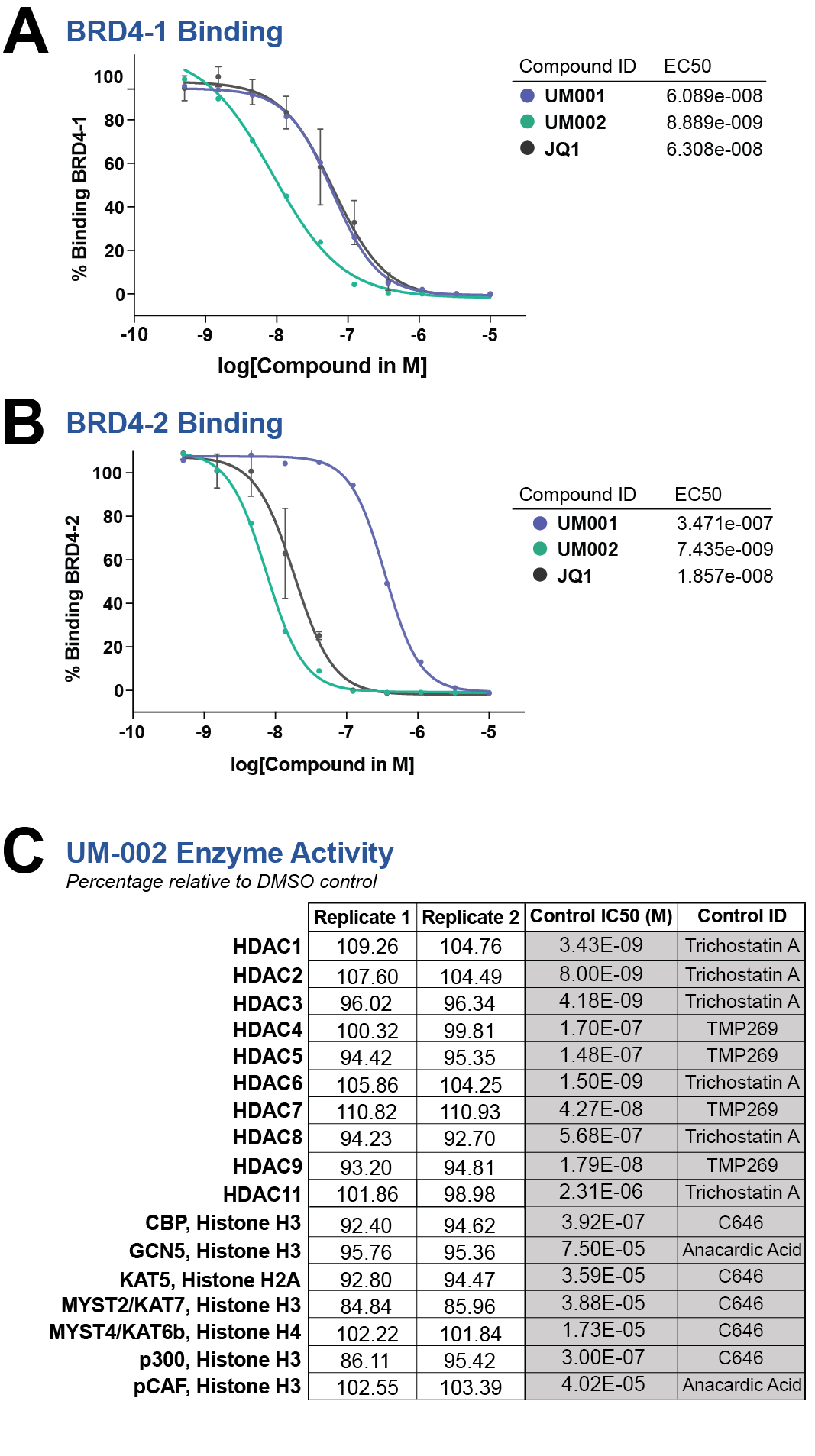


**Supplementary Figure S1: UM-002 does not inhibit HDACs and HATs. A-B.** BRD4-1 and BRD4-2 binding assays for UM-002 and JQ1. These data are an average of two replicates and regression curves were plotted in GraphPad PRISM (version 8.4.3). **C.** Percentage activity for UM-002 relative to DMSO against a panel of HDACs and HATs.

**
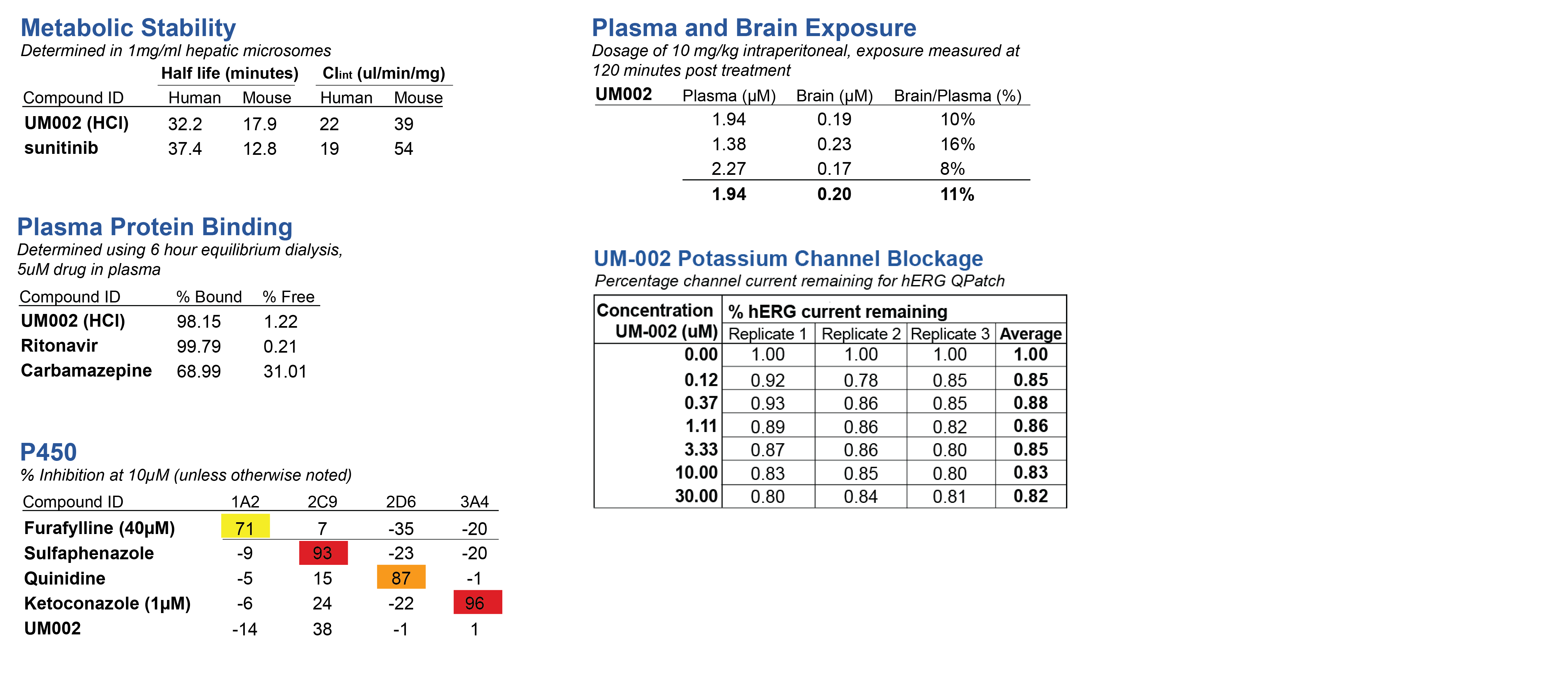
**

**Supplementary Figure S2: UM-002 is brain penetrant.** Left Panel: Metabolic Stability, Plasma Protein Binding, and P450 inhibition assays for UM-002. Right Panel: Plasma and Brain Exposure measured *in vivo,* and Potassium Channel Blockage measured by hERG QPatch.

**
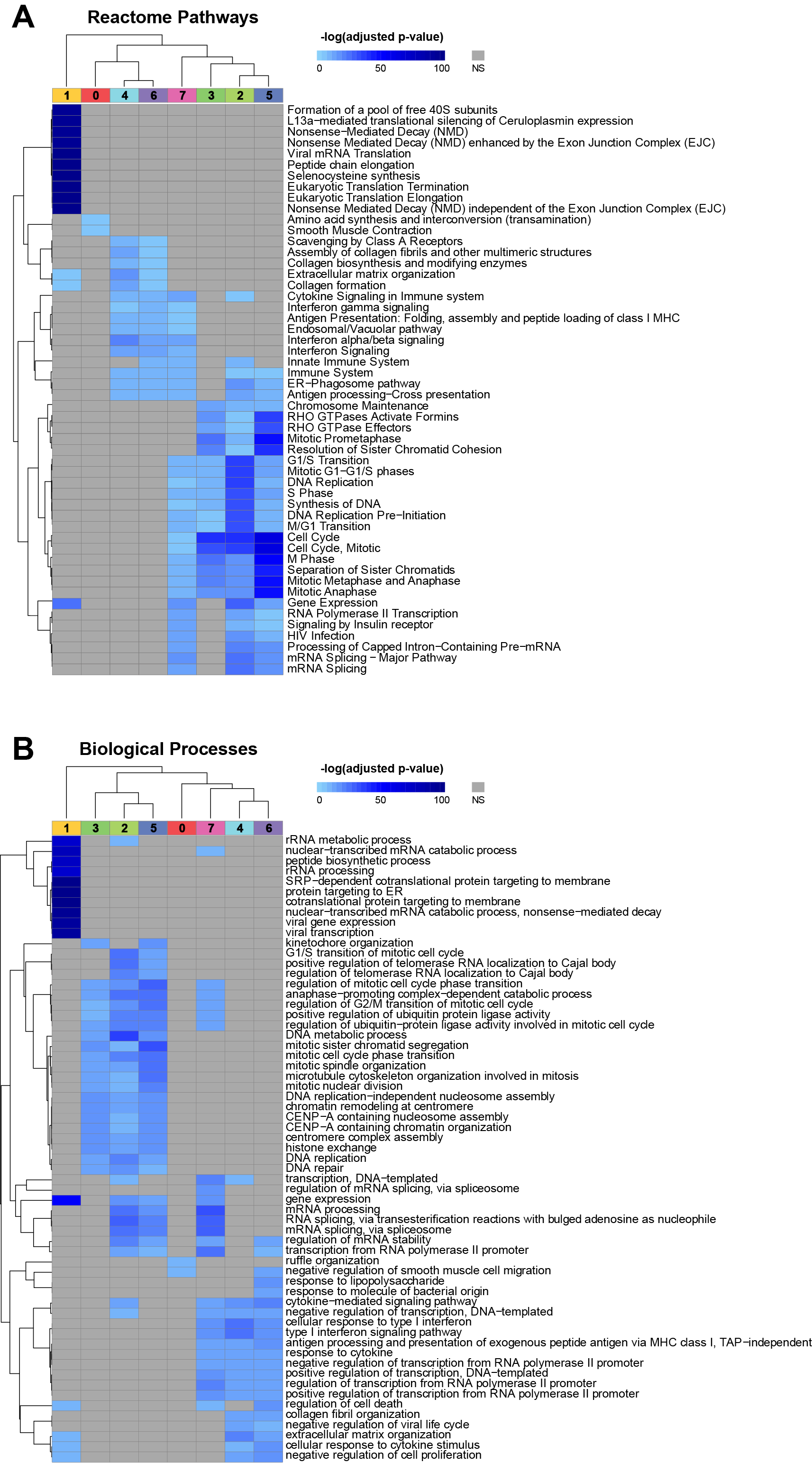
**

**Supplementary Figure S3: Pathway and gene ontology enrichment characterizes distinct GBM22 clusters based on invasion and cell cycle state.** Significantly upregulated genes (average log_2_ fold change > 0.25) from each SNN cluster of G22 cells as defined in Figure 6 were analyzed for gene ontology and pathway analysis. Top ten significantly enriched (adjusted p-value < 0.05) terms were identified for each cluster and plotted in a heatmap for Reactome pathways **(A)** and Gene Ontology Biological Processes **(B)**. Pathway analysis was performed using Enrichr (<https://maayanlab.cloud/Enrichr/>) and heatmaps were generated using the R package pheatmap (version 1.0.12; https://CRAN.R-project.org/package=pheatmap).

**Supplementary Table S1.** **UM-002 inhibits BET bromodomain proteins.** Differential sensitivity for bromodomain proteins was assessed using an AlphaScreen binding assay. Compounds were tested with 3-fold serial dilutions starting at 10 μM and percentage binding was calculated relative to DMSO control. IC50 values were computed using nonlinear regression (variable slope, 4 parameter) fitting in GraphPad Prism 8 (version 8.4.3).

| **Bromodomain** | **UM-002 IC50 (M)** | **Control IC50 (M)** | **Control** |
| --- | --- | --- | --- |
| BRD1 | 2.55E-08 | ~8.66E-05 | Bromosporine |
| BRD2-1 | 1.27E-08 | 7.06E-08 | JQ1 |
| BRD2-2 | 7.12E-09 | 1.42E-08 | JQ1 |
| BRD3-1 | 8.38E-09 | 2.21E-08 | JQ1 |
| BRD3-2 | 2.55E-08 | 3.4E-08 | JQ1 |
| BRD4-1 | 8.89E-09 | 6.31E-08 | JQ1 |
| BRD4-2 | 7.44E-09 | 1.86E-08 | JQ1 |
| BRD9 | 7.64E-07 | 2.34E-07 | Bromosporine |
| BRDT-1 | 1.43E-08 | 8.78E-08 | JQ1 |

**Supplementary Table S2: UM-002 altered the expression of GBM relevant genes *in vivo.*** Genes of interest from Figure 7B were evaluated for relevance to GBM and correlation with survival. Survival was assessed using the TCGA_GBM dataset in Gliovis (http://gliovis.bioinfo.cnio.es/), with a cutoff of high versus low gene expression.

| **Gene** | **Clusters** | **FC** | **GBM Relevance** | **TCGA Survival Correlation** |
| --- | --- | --- | --- | --- |
| MFAP5 | 0 | -0.23, padj 3.88E-16 | EMT^48^ | Log-rank p 0.64 |
| S100A4 | 0  1  2  3  4  5 | -0.36, padj 2.96E-51  -0.48, padj 1.69E-49  -0.34, padj 4.36E-27  -0.33, padj 1.51E-19  -0.31, padj 1.32E-15  -0.35, padj 4.52E-14 | EMT^49^ | Log-rank p 0.25 |
| RRAD | 0  1 | -0.25, padj 2.40E-13  -0.43, padj 7.87E-22 | Therapy Resistance^50^ | **HR = 0.57, (0.33 - 0.96)**  **Log-rank p 0.03*** |
| SEC61G | 0  1  2 | -0.27, padj 3.45E-72  -0.32, padj 3.52E-57  -0.24, padj 5.31E-61 | Therapy Resistance^51^ | Log-rank p 0.28 |
| ANXA1 | 0  1 | -0.25, padj 2.80E-55  -0.27, padj 1.05E-22 | Invasion^52^ | Log-rank p 0.06 |
| SPARC | 1 | -0.24, padj 5.21E-10 | Invasion^53-55^ | Log-rank p 0.32 |
| PRDX1 | 1 | -0.24, padj 1.22E-17 | Therapy Resistance^56^ | Log-rank p 0.99 |
| AKR1B1 | 1 | -0.25, padj 2.56E-16 | Survival^57^ | Log-rank p 0.17 |
| TMSB4X | 1 | -0.30, padj 5.82E-30 | Invasion^58^ | unavailable |
| IGFBP7 | 4 | -0.31, padj 9.83E-12 | Migration/Invasion^59^ | Log-rank p 0.97 |
| FXYD5 | 5 | -0.24, padj 1.09E-14 | Invasion, EMT^60^ | **HR = 0.59, (0.35 – 1)**  **Log-rank p 0.045*** |
| RBM3 | 5 | -0.25, padj 1.27E-11 | Proliferation and Invasion^61^ | Log-rank p 0.69 |
| KDELR2 | 1 | -0.26, padj 5.18E-28 | Tumorigenesis^62^ | **HR = 0.52, (0.31 – 0.85)**  **Log-rank p 0.008*** |
| PLAT | 1 | -0.28, padj 1.66E-15 | Stemness^63^ | Log-rank p 0.095 |
| ARF4 | 1 | -0.25, padj 5.91E-24 | Anti-Apoptotic^64^ | Log-rank p 0.41 |
| PVT1 | 0  2  3 | 0.44, p-adj 3.45E-72  0.47, p-adj 1.52E-34  0.35, p-adj 1.77E-17 | Proliferation & Invasion^65^ | Log-rank p 0.44 |
| VEGFA | 1 | 0.31, p-adj 1.84E-10 | Angiogenesis & Invasion^66^ | Log-rank p 0.12 |
| NDRG1 | 1 | 0.31, p-adj 9.28E-16 | Proliferation & Angiogenesis^67^ | **HR = 0.48, (0.29 – 0.82)**  **Log-rank p 0.006*** |
| ZFAS1 | 1 | 0.24, p-adj 1.57E-37 | Proliferation & Invasion^68^ | unavailable |
| CEBPB | 1 | 0.31, p-adj 2.55E-15 | Mesenchymal marker^69^ | Log-rank p 0.15 |
| KRT14 | 1 | 0.25, p-adj 8.25E-10 | Epithelial marker^70^ | Log-rank p 0.17 |
| ARL4C | 1 | 0.39, p-adj 5.76E-13 | Invasion^71^ | **HR = 0.46, (0.26 – 0.8)**  **Log-rank p 0.005*** |
